# Molecular Disorganization of Axons Adjacent to Human Cortical Microinfarcts

**DOI:** 10.3389/fneur.2017.00405

**Published:** 2017-08-16

**Authors:** Hamza Coban, Spencer Tung, Bryan Yoo, Harry V. Vinters, Jason D. Hinman

**Affiliations:** ^1^Department of Pathology and Laboratory Medicine, Section of Neuropathology, David Geffen School of Medicine, University of California Los Angeles, Los Angeles, CA, United States; ^2^Department of Radiology, Division of Neuroradiology, David Geffen School of Medicine, University of California Los Angeles, Los Angeles, CA, United States; ^3^Department of Neurology, David Geffen School of Medicine, University of California Los Angeles, Los Angeles, CA, United States

**Keywords:** microinfarct, cortical microinfarct, small vessel disease, neuropathology, vascular dementia

## Abstract

Cortical microinfarcts (CMIs) are microscopically identified wedge-shaped ischemic lesions that occur at or near the cortical surface and result from occlusion of penetrating arterioles. These microscopic lesions can be observed with high-resolution magnetic resonance imaging in aging brains and in patients with cerebrovascular disease. Recent studies have suggested that strategically located microinfarcts strongly correlate with cognitive deficits, which can contribute to Alzheimer’s disease as well as other forms of dementia. We have recently shown that the molecular organization of axons into functional microdomains is altered in areas adjacent to white matter lacunar and microinfarcts, creating a peri-infarct penumbral injury in surviving axons. Whether similar changes in nodal, adjacent paranodal, and proximal axon initial segment molecular organization occur in the cortex adjacent to human CMIs is not known. Paraffin-embedded sections of autopsy brain tissue from five patients with CMIs were immunofluorescently labeled for nodal and paranodal markers including beta-IV spectrin, ankyrin-G, and contactin-associated protein. High magnification images from the peri-infarct cortical tissue were generated using confocal microscopy. In surviving cortical tissue adjacent to microinfarcts, we observed a dramatic loss of axon initial segments, suggesting that neuronal firing capacity in adjacent cortical tissue is likely compromised. The number of identifiable nodal/paranodal complexes in surviving cortical tissue is reduced adjacent to microinfarcts, while the average paranodal length is increased indicating a breakdown of axoglial contact. This axonal microdomain disorganization occurs in the relative absence of changes in the structural integrity of myelinated axons as measured by myelin basic protein and neurofilament staining. These findings indicate that the molecular organization of surviving axons adjacent to human CMIs is abnormal, reflecting lost axoglial contact and the functional elements necessary for neural transmission. This study provides support for the concept of a microinfarct penumbral injury that may account for the cumulative cognitive effect of these tiny strokes.

## Introduction

The pathophysiologic cause(s) of cognitive impairment leading to dementia remains unclear. Accumulations of amyloid-beta, neurofibrillary tangles with hyperphosphorylated tau, selective neuronal loss, as well as poorly classified ischemic brain lesions are all implicated, yet appear to incompletely explain the pathophysiology. Recent studies suggest that cerebral microinfarcts, in particular, cortical microinfarcts (CMIs), are another type of brain injury related to cognitive decline and dementia syndromes. In part because of their small size, comparatively little is known about how these lesions might contribute to cognitive decline and dementia. Cerebral microinfarcts are not visible in current neuroimaging resolution limits, only recognized on microscopic examination (1). Even with the ultra-high-resolution 7.0 T magnetic resonance imaging (7 T MRI), only lesions larger than 0.5 mm can be visualized *in vivo* (2), and current clinically utilized MRI protocols will miss nearly all CMIs (1). The size of a microinfarct is generally defined as “not visible with the naked eye” or “only visible upon light microscopy,” but the size of a microinfarct is generally accepted as being 50–400 μm and described as gliotic or cystic lesions with neuronal loss and tissue pallor that may occur in all brain regions, although the cerebral cortex may be a preferential location (3). Thus, detailed pathologic analysis of these lesions is critical to understanding how they contribute to cognitive decline.

Cortical microinfarcts are present in 6.3–30% of aging brains and even more commonly in those with dementia (36.5%) (4, 5). CMIs are associated with both subtle signs of cognitive decline and dementia (6). Moreover, these small lesions also increase the risk for recurrent ischemic and hemorrhagic stroke (7). Although the importance of CMIs in cognitive impairment, dementia, and overall brain health is increasingly recognized, the underlying pathophysiology of CMIs is not completely understood. Recent efforts to model these lesions in rodents suggest that in part their effect is cumulative and results from a decrease in regional neuronal network activity (8). In part, the cumulative effect of CMIs may be a consequence of expanded regional injury in the form a penumbral field where damage to axons of surviving neurons impairs neuronal connectivity. Building on prior work that demonstrated alterations of the molecular organization of axons in regions adjacent to human white matter lacunar and microinfarcts (9), in this study, we hypothesized that similar changes were present in nodal, paranodal, and axon initial segments (AISs) adjacent to human CMIs. The findings suggest that CMIs are indeed associated with an expanded region of impaired axonal microdomain organization with a significant loss of AISs in surviving neurons and disruptions of adjacent nodal and paranodal segments in cortical regions adjacent to the microinfarct. This supports the concept of a penumbral region adjacent to human CMIs in which progressive neuronal and axonal injury could be blunted to mitigate the effect of CMIs on cognitive impairment.

## Materials and Methods

### Clinical Case Selection

We retrospectively reviewed the medical records and neuropathology reports of 64 autopsy patients at the University of California, Los Angeles Mary E. Easton Alzheimer’s Disease Research Center Neuropathology Core Brain Bank between January 1, 2013, and December 31, 2014. Written informed consent for autopsy was obtained from all subjects or legal next-of-kin. According to University California Los Angeles policy, deidentified data from autopsy materials is not subject to ethics committee approval. Pathologic assessments were performed by neuropathologists at the University of California, Los Angeles Department of Pathology and Laboratory Medicine, Division of Neuropathology. Patients with known neoplasms thought to be associated with the neurologic symptoms and those with immunodeficiency, such as HIV infection and transplant patients, were excluded from the study. Individuals younger than 50 years or with a history of intractable epilepsy undergoing focal cortical excision for diagnostic or therapeutic purposes were also excluded. From this cohort, 18 cases with dementia were selected, which include the term “microinfarct” in their neuropathology reports for detailed microscopy for definable CMIs determined by expert neuropathologist (Dr. Harry V. Vinters) assessment on hematoxylin and eosin-stained sections. After evaluation of these 18 cases, 5 of them were identified with definable CMIs. Control cases were selected from the same cohort. After same exclusion criteria applied, age-matched two cases were identified without clinical or neuropathologic diagnosis of dementia. Demographic information and anatomical location of infarcts for each case are detailed in Table 1. Based on the clinical information available at autopsy, all the lesions identified were presumed to be asymptomatic in life.

**Table 1:** Clinical demographics.

Case ID	Age (years)	Sex	Region examined	Infarct size (mm^2^)	Post-mortem interval (h)	Comorbidities
Subject-1	75	M	Right occipital	0.306	33	Alzheimer’s disease, coronary artery disease
Subject-2	86	M	Right occipital	0.481	10	Dementia, prostate cancer, coronary artery disease
Subject-3	84	M	Right occipital	0.457	4	Parkinson’s disease, frontotemporal dementia
Subject-4	93	F	Right frontal	0.891	4	Alzheimer’s disease, cerebrovascular disease
Subject-5	73	M	Right frontal	0.961	45	Parkinson’s disease, dementia, hypertension
Control-1	83	M	Right occipital	N/A	19	Coronary artery disease
Control-2	72	F	Right frontal	N/A	24	Congestive heart failure, diverticulosis

*The age, sex, infarct location, infarct size, post-mortem intervals, and medical comorbidities of each subject. Cases were selected for detailed microscopy included those with definable microinfarcts determined by expert neuropathological assessment of hematoxylin and eosin-stained sections*.

### Tissue Processing and Immunohistochemistry

Autopsy and brain extractions were performed according to standard procedures. Post-mortem intervals are detailed in Table 1. Extracted brains were immediately fixed in 10% neutral buffered formalin for at least 2 weeks. Fixed specimens were blocked for regions of interest, processed, and embedded in paraffin. Six-micron-thick tissue sections were generated from the paraffin-embedded blocks for histological analysis using immunofluorescent labeling as follows. Sections were baked at 60°C to melt the paraffin wax and subsequently washed in xylene to dissolve residual paraffin followed by graded ethanol baths (100–70%) and double-distilled H_2_O to rehydrate the tissue. For histological staining, sections were then immersed in Harris Hematoxylin (Fisher) for 4 min and slightly destained in 1% HCl in 80% ethanol. Hematoxylin-stained slides were further developed with 0.2% ammonia water and then immersed in Eosin-Y (Fisher) for 3 min. Following eosin immersion, sections were dehydrated in graded ethanol baths (70–100%), cleared in xylenes, and cover-slipped for visualization by light microscopy. For immunofluorescent labeling, tissue epitopes were exposed with heat-induced antigen retrieval using a pressurized antigen decloaking chamber at 120°C for 5 min in 10 mmol citrate pH 6.0 and allowed to cool to room temperature. After antigen retrieval, tissue sections were permeabilized with 0.3% TritonTM X-100 in phosphate-buffered saline (PBS). After permeabilization, sections were blocked with 5% normal donkey serum 0.3% TritonTM X-100 in PBS (NDSX) for 1 h. Finally, sections were incubated overnight sequentially with the following primary antibodies: mouse anti-contactin-associated protein (caspr) (1:500, Neuromab), rabbit anti-Ankyrin-G (Ank-G) (1:150, gift from Dr. Matthew Rasband, Baylor College of Medicine), mouse anti-human myelin basic protein (MBP) (1:1,000, Chemicon) and rabbit anti-neurofilament 200 (NF200) (1:500, Sigma) in NDSX, visualized using appropriate immunoflourescent anti-mouse (Invitrogen A21202), and anti-rabbit secondary (Invitrogen A10042) antibodies diluted at 1:200 in NDSX and mounted in medium containing DAPI for nuclear counterstaining (Invitrogen P36935).

### Imaging and Quantification

Histologic sections stained for hematoxylin and eosin were imaged on a Leica DMLB microscope. CMIs were identified as cavitary or elliptical lesions with loss of tissue, tissue pallor, neuronal loss with few remaining macrophages, and surrounding fibrillary gliosis. Microinfarct areas were measured using ImageScope^®^ version 12.1.0.5029 (Aperio Inc.) (Table 1). A center point of each lesion was identified, and the horizontal and vertical diameters were measured. Immunolabelled serial sections were imaged at ×60–100 using a Nikon C2 laser scanning confocal microscope. The region containing the microinfarct was co-registered with the hematoxylin and eosin staining and six high-powered microscopic fields were imaged that corresponded to the peri-infarct regions within 200 µm of the microinfarct. Measurements were made by ImageJ 1.49v (national Institute of Health, USA). For nodal and paranodal measurements, only axons with an identifiable node and two adjacent paranodes were measured to avoid measuring axons that were cut during thin sectioning. Control measurements were determined using both internal control (obtained by measuring segments from regions of cortex away from the region of microinfarct) and external controls from age-matched tissue samples without evidence of infarct.

## Results

To determine the molecular organization of axons adjacent to CMIs, we selected 5 clinical cases with pathologically identifiable microinfarcts from a cohort of 18 cases in which the neuropathology report included the term “microinfarct.” This reflects an incidence rate of CMIs of 28.1% (18/64) in the larger patient cohort available for review. Age-matched control subjects were identified from the neuropathology database available at our institution. The age, sex, infarct location, post-mortem interval, infarct size, and medical comorbidities of each case are presented in Table 1. The patients selected ranged in age from 72 to 93 years. All of the subjects carried a pre-mortem diagnosis of dementia.

**Figure 1:**
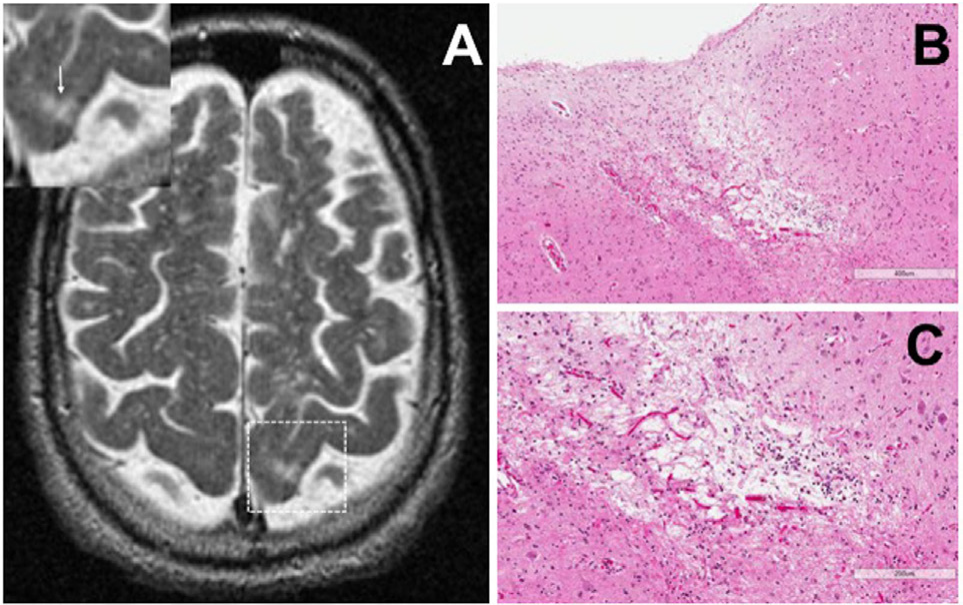
Representative axial T2-weighted magnetic resonance imaging (MRI) image from one case included in the study. The MRI image demonstrates partial CSF-signal intensity of a cortical microinfarct in the left occipital cortex (arrow in the inset) **(A)**. Hematoxylin and eosin-stained sections taken from the left occipital cortex show the classic features of a cortical microinfarct marked by a small localized necrotic core immediately adjacent to the cortical surface [**(B,C)**; ×10 and ×20 magnification, respectively].

**Figure 2:**
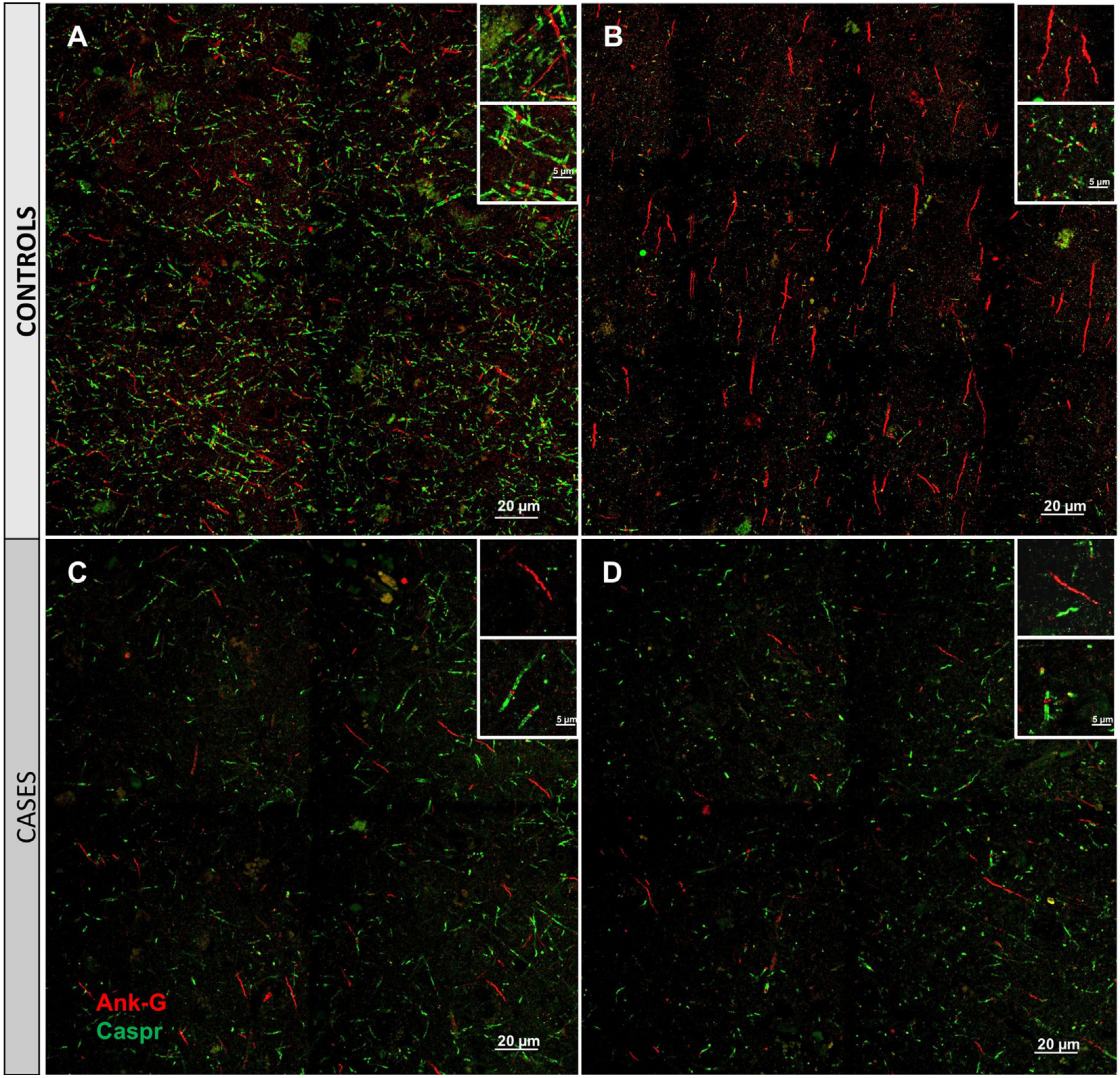
Immunofluorescent labeling for nodal and axon initial segment (AIS) marker ankyrin-G (Ank-G, red) and paranodal marker contactin-associated protein (caspr, green) in controls **(A,B)** and cases **(C,D)**. Labeling of AIS and nodal and paranodal axonal microdomains in cortical tissue from control cases reveals a regular pattern multiple intact node/paranode complexes **(A)** and multiple AISs **(B)**. In cortical tissue adjacent to a microinfarct from two different cases **(C,D)**, the frequency of intact AIS is dramatically reduced. Similarly, intact node/paranode complexes are rarely identified and when present often show paranodal elongation [inset **(C)**]. Inset: magnification ×60, scale bar = 5 µm.

**Figure 3:**
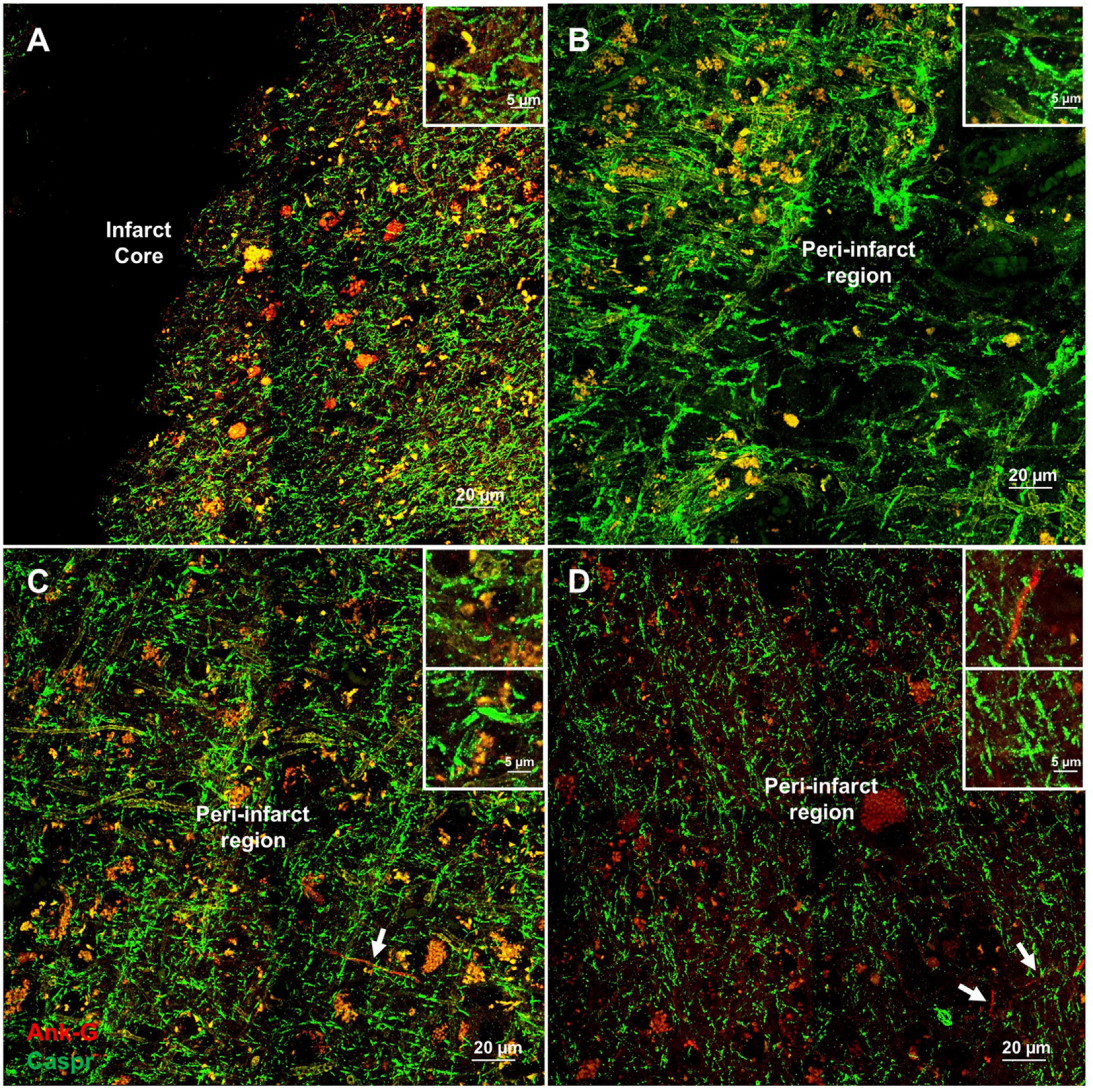
Additional examples of axonal microdomain disruption are apparent immediately adjacent to microinfarcts. Elongated and irregular contactin-associated protein (caspr)-positive paranodes (green) are frequently seen **(A)**. In tissue adjacent to, but some distance from the infarct core (300–350 µm away from infarct core), axonal microdomains remain disrupted with irregular morphology of paranodal regions [inset **(B)**]. In some instances, Ankyrin-G (Ank-G)-positive axon initial segments can be identified [arrowheads, insets **(C,D)**]. Inset: magnification ×60, scale bar = 5 µm.

Cortical microinfarcts were defined pathologically by the presence of a focal necrotic cystic cavity by hematoxylin and eosin staining. In selected cases, clinically available MRI scans were reviewed, and the lesion was visible and therefore co-registered with the pathologic specimen (Figure 1). Hematoxylin and eosin staining of each of the CMIs studied is presented in Figure S1 in Supplementary Material, and each demonstrate the classic hallmarks of infarction with a necrotic core, tissue pallor, and an elliptical or wedge-shaped pattern consistent with infarction secondary to occlusion of a penetrating arteriole from the cortical surface. The area (in square millimeter) of each defined microinfarct was measured and is presented in Table 1. All of the infarcts were less than 1 mm^2^ in area. To examine the peri-infarct tissue in a systematic way that would account for potential changes at different cortical depths, we identified six peri-infarct regions of interest adjacent to each microinfarct measuring 375 µm^3^ (Figure S2 in Supplementary Material). Axonal microdomains were immunolabeled for Ank-G to identify nodes of Ranvier and AISs as well as caspr to label paranodes. In age-matched control cortex, normal labeling of nodes and paranodes (Figure 2A) and robust labeling of AISs within corresponding regions of cortex was observed (Figure 2B). In cortical tissue adjacent to microinfarcts, AISs could be identified (Figures 2C,D; upper insets) but were decreased in density by 87% compared to controls (*p* = 0.008 using Student’s *t*-test, Welch’s correction; Table 3). The number of node/paranode complexes identifiable in cortical tissue was similarly decreased by 86.2% compared to controls (Figures 2C,D; Table 2). Closer examination of the peri-infarct regions adjacent to CMIs demonstrated profound disorganization of paranodal segments in tissue both immediately adjacent to the infarct and in peri-infarct tissue 100–200 µm away from the infarct core (Figure 3). Very few recognizable node/paranode complexes could be identified in peri-infarct tissue. Beyond this pattern of disorganization, a frequent finding included paranodal segments with “empty” nodes that were not stained for Ank-G (inset, Figures 3B,C). Occasional AISs were identifiable in peri-infarct tissue but were rare (Figures 3C,D). In regions of cortical tissue away from the microinfarct, we observed typical patterns of nodal, paranodal, and AIS staining, indicating that these effects were specific to the peri-infarct regions adjacent to CMIs.

**Table 2:** Statistical analysis.

Cumulative average	Number of axon initial segment (AIS)	SD	*p*-Value	Number of N/PN complex	SD	*p*-Value
Subjects (*n* = 5)	5.8	4.6583	0.0078	14	11.5542	0.1687
Controls (*n* = 2)	44.5	4.9497	n/a	102	73.5391	n/a

*Regions of cortex adjacent to microinfarcts demonstrated multiple irregularities. Number of AISs were significantly decreased by 87% in average number of AIS when compared to controls (*p* = 0.0078). Number of node–paranode complex was also decreased by 86.2% in average number of node–paranode complex when compared to controls (*p* = 0.1687)*.

**Table 3:** Statistical analysis.

Cumulative average	Axon initial segment (AIS) length (µm)	SD	*p*-Value	PN length (µm)	SD	*p*-Value
Subjects (*n* = 5)	12.768	4.3469	0.3328	2.9712	0.5582	0.0769
Controls (*n* = 2)	11.276	0.0834	n/a	2.0765	0.8831	n/a

*In the regions adjacent to the microinfarct, AISs were disrupted with a 21.7% increase in average AIS length when compared to control (*p* = 0.3328). There was no difference in AIS length in the remaining tissue. Paranodal segments were also disrupted with a 30.2% increase in average paranodal length when compared to control (*p* = 0.0769)*.

A key determinant of the functionality of the AISs is segment length, which correlates with firing potential (10, 11). Therefore, we measured AIS length in remaining AISs adjacent to CMIs. There was a modest increase in AIS length by 21.7% although this was not significantly differently from control. Similarly, paranodal length has been shown to be an indicator of axoglial injury (9, 12–14). The length of measured paranodes within the cortical tissue adjacent to CMIs demonstrated at 30.2% increase in length compared to controls (*p* = 0.077 using Student’s *t*-test, Welch’s correction; Table 3). The disorganized pattern of caspr staining within the peri-infarct regions with frequent “empty” nodes complicates this analysis, as we limited our paranodal length measurements to node–paranode complexes in which both staining elements were positive, so as to limit the effect of measuring paranodal segments affected by tissue sectioning.

**Figure 4:**
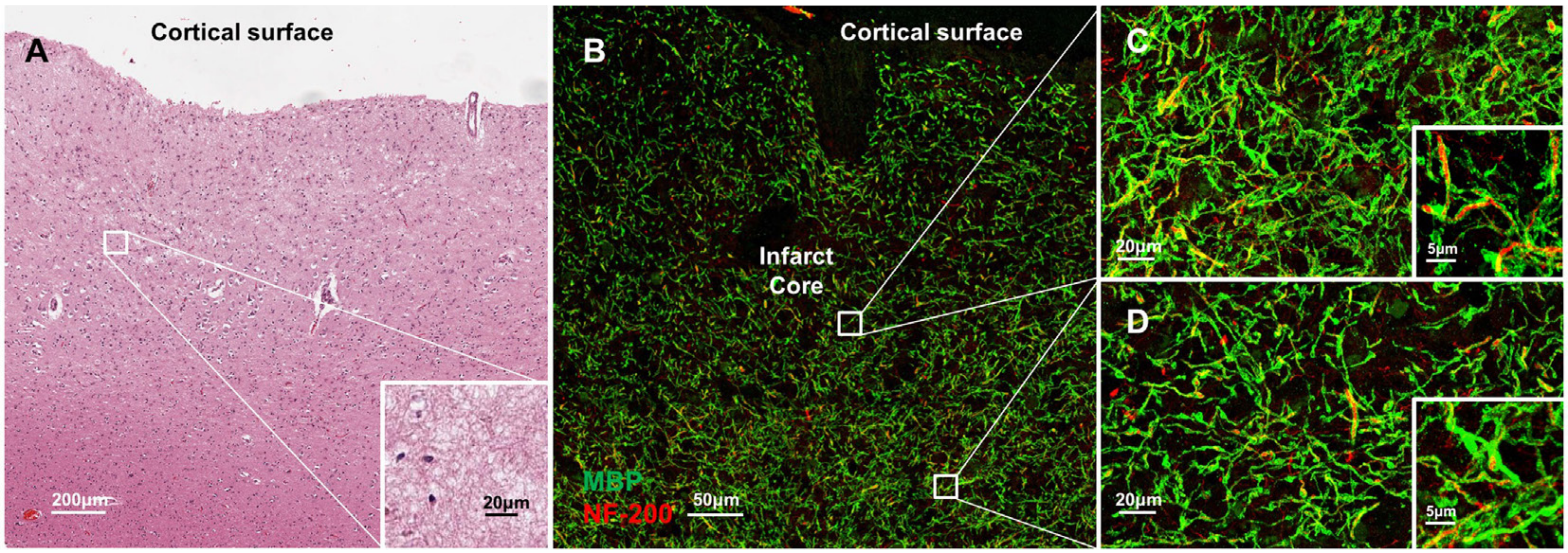
Molecular disorganization of axons adjacent to microinfarct exists in the absence of significant changes in structural integrity of myelin and axons. Cortical tissue containing a microinfarct **(A)** stained for myelin basic protein (MBP, green) and neurofilament 200 (NF200, red) demonstrates very few demyelinated axons adjacent to the infarct core **(B)**. Co-localization of MBP and NF200 indicates preservation of the structural organization of myelinated axons in regions both immediately adjacent to the infarct core **(C)** as well as some distance from the infarct core where disrupted axonal microdomain organization was observed **(D)**. Inset: magnification ×60, scale bar = 5 µm.

Molecular disorganization of specific proteins within the axon, such as ankryin G or caspr, may reflect loss of myelin or other structural disruption of the axons (15–19). To examine the structural integrity of myelin and axons adjacent to CMIs, we labeled serial sections from each case for MBP and NF200 (Figure 4). While there is some evidence of denuded axons immediately adjacent to the infarct core (Figure 4B), high magnification images at increasing distances from the infarct core (Figures 4C,D), where we had examined axonal microdomain organization, revealed relative preservation of myelin and axons as evidenced by the close co-localization of MBP and NF200 staining.

## Discussion

The relationship between cerebrovascular disease (CVD) and Alzheimer’s pathology has become increasingly complex. Careful pathologic inspection of sporadic cases of Alzheimer’s disease brains reveals microinfarcts including the presence of CMIs as a frequent finding (20). The extent to which these tiny lesions contribute to the cognitive dysfunction of dementia remains unknown. Here, we show that within the surviving cortical tissue immediately adjacent to CMIs, the functional molecular organization of axons is disrupted. Penumbral tissue adjacent to CMIs is marked by a profound loss of AISs, a loss of node/paranode complexes, and “empty” nodal domains. These changes occur in the absence of changes in the basic structure of myelinated axons within the penumbral region. These findings provide support for the concept of a microinfarct penumbra that can extend beyond the focal loss of neurons associated with the primary infarct to jeopardize cortical connectivity. Moreover, they provide further evidence that axoglial interactions are exquisitely sensitive to ischemic injury and fertile ground for the development of neural repair strategies targeting axonal regeneration (21).

Advances in high-resolution neuroimaging studies have resulted in increasing recognition of the many manifestations of CVD that go well beyond classic multi-infarct dementia (22). These include cortical, white matter, and deep nuclei microinfarcts detected on high-resolution structural imaging as well as disruptions of white matter connectivity evidenced by diffusion tensor imaging (6, 23). This more complete spectrum of CVD and its pathologic correlates demand greater attention to the neurobiologic substrates that result from these injuries. Recent studies have shown that unlike cortical microhemorrhages, which could be partially detected using routine clinical MRI (1.5 T), CMIs were uniformly not detected and only identifiable on pathologic examination of brain specimens (1). Such pathologic specimens therefore provide a unique opportunity to improve our understanding of the effect of these lesions. Furthermore, the identification of therapeutics targeting repair of these lesions necessitates knowledge of the molecular events occurring around them. Notably, the 200-µm peri-infarct region examined in this study correspond exactly to the peri-infarct region most active in axonal sprouting and neural repair after focal cortical stroke in rodents (24–27). Animal modeling of CMIs has demonstrated support for a micropenumbral region adjacent to CMIs with the occlusion of multiple neighboring penetrating arterioles leading to microinfarct enlargement (8), indicating that the immediately adjacent cortical tissue is vulnerable to additional injuries with the axon being particularly vulnerable to partial injury.

The AIS is a complex and unique axonal microdomain that essentially functions as an on–off switch for the neuron (28). At the AIS, dendritic and cell body input signals are integrated and modulated by axo-axonic synapses to control the firing rate of an individual neuron. Diminishing the length of the AIS *in vivo* results in fewer voltage-gated sodium channels at the AIS and therefore a higher firing threshold (29). Ank-G has been shown to be the critical regulator of AIS formation (30), and acting together with beta-IV spectrin, it functions to anchor voltage-gated sodium channels (Na_V_) to the AIS membrane (31). In rodents, the AIS has shown exquisite sensitivity to direct ischemic injury that in part is driven by sensitivity to the activation of calpain (32). In this context, AIS loss in the penumbral regions of cortical tissue adjacent to human CMIs is not surprising. This study is the first to report such sensitivity of the AIS to partial ischemia in humans. In severe brain ischemia, calpain activation results in degradation of the structural elements of the axon including neurofilaments (33); however, we did not observe evidence of significant axonal degeneration in micropenumbral regions adjacent to CMIs nor did we observe complete absence of AIS in these regions. This suggests a more subtle disruption of the AIS in penumbral neurons adjacent to microinfarcts and further highlights the selective vulnerability of some cortical neurons.

Myelinated axons are predominantly associated with white matter, although in lesser density are readily identifiable throughout the cortex with some regional variation and increases in density in lower cortical layers. Here, we show the presence of mostly myelinated axons in the cortex, although smaller unmyelinated axons may be lost during post-mortem intervals and during tissue processing. They may also not stain robustly with NF200 and therefore be below the level of detection of this staining and imaging approach. Regardless, the function of myelinated axons is to speed neural transmission by clustering Na_V_ at nodes of Ranvier facilitating saltatory conduction. Disruption of the normal molecular organization of axons at nodal and paranodal regions is associated with axoglial pathologies including ischemia (34, 35). This disruption primarily results in the loss of nodal components and lengthening of paranodes due to lost cell–cell adhesion between oligodendrocytes and axons as we observed adjacent to CMIs. The observed decrease in nodal/paranodal complexes in the absence of significant structural changes in myelinated axons indicates that reliable, high-fidelity axonal conduction in cortical tissue adjacent to CMIs is not possible. Similarly, paranodal lengthening within penumbral regions indicates that axoglial interactions at the paranode are jeopardized in these regions, implying their sensitivity to partial ischemia. Modifications of the extracellular matrix after cortical ischemia may play an important role in stabilizing axoglial interactions (36). A limitation of this post-mortem human study is that because this peri-infarct region is known to be an active participant in axonal sprouting and neural repair, altered axoglial interactions could reflect incomplete myelination of newly sprouted axons. The phenomena of peri-infarct myelination during ischemic recovery deserves further study with animal models; however, these authors favor that the observed changes in axonal microdomain organization at nodes and paranodes adjacent to CMIs reflect an injury signal rather than evidence of a partial reparative phenomena.

The emergence of CMIs as a contributor to cognitive dysfunction in dementia and a previously underrecognized manifestation of cerebral microvascular disease highlights the need to understand the pathology of these lesions in the context of known principles of neural repair after stroke. A penumbral region adjacent to CMIs marked by neurons with diminished firing potential and disrupted axonal communication may exacerbate the cognitive effect of these tiny cerebrovascular lesions. Further identification of the molecular events active within this penumbral region may help to generate novel therapeutic strategies to repair the brain after cortical microinfarction.

## Author Contributions

HC: had conducted this project and drafted the manuscript under the supervision of JH. ST: assisted HC in doing immunohistochemical and immunofluorescence stainings. HV: contributed to this project with his expertise in neuropathology particularly identifying lesions and case selection and he also edited the manuscript. BY: reviewed the MRI images of the subjects. JH: supervised the project from beginning to drafting and editing the manuscript.

## Conflict of Interest Statement

The authors declare that the research was conducted in the absence of any commercial or financial relationships that could be construed as a potential conflict of interest.

## Supplementary Material

Figure S1 Hematoxylin–eosin stained sections of cortical layers from four patients involved in the study. ×10 magnification.

Figure S2 Illustration of a watershed cortical area from which detailed analysis was undertaken. Pink area indicates watershed cortical region with penumbra, and framed areas indicate one-unit area of the sites of the images taken for analysis. Six different areas were examined with equal distance from the infarct core. 1 unit area = 250 µm × 250 µm.

## References

[1] van Veluw SJ Charidimou A Van Der Kouwe AJ Lauer A Reijmer YD Costantino I Microbleed and microinfarct detection in amyloid angiopathy: a high-resolution MRI-histopathology study. Brain (2016) 139(Pt 12):3151–62. 10.1093/brain/aww229 27645801 PMC5840880

[2] van Veluw SJ Zwanenburg JJ Engelen-Lee J Spliet WG Hendrikse J Luijten PR In vivo detection of cerebral cortical microinfarcts with high-resolution 7T MRI. J Cereb Blood Flow Metab (2013) 33:322–9. 10.1038/jcbfm.2012.196 23250109 PMC3587820

[3] Brundel M De Bresser J Van Dillen JJ Kappelle LJ Biessels GJ. Cerebral microinfarcts: a systematic review of neuropathological studies. J Cereb Blood Flow Metab (2012) 32:425–36. 10.1038/jcbfm.2011.200 22234334 PMC3293128

[4] Kovari E Gold G Herrmann FR Canuto A Hof PR Michel JP Cortical microinfarcts and demyelination significantly affect cognition in brain aging. Stroke (2004) 35:410–4. 10.1161/01.STR.0000110791.51378.4E 14707236

[5] Arvanitakis Z Leurgans SE Barnes LL Bennett DA Schneider JA. Microinfarct pathology, dementia, and cognitive systems. Stroke (2011) 42:722–7. 10.1161/STROKEAHA.110.595082 21212395 PMC3042494

[6] Hilal S Sikking E Shaik MA Chan QL Van Veluw SJ Vrooman H Cortical cerebral microinfarcts on 3T MRI: a novel marker of cerebrovascular disease. Neurology (2016) 87(15):1583–90. 10.1212/WNL.0000000000003110 27590296

[7] Wilson D Charidimou A Ambler G Fox ZV Gregoire S Rayson P Recurrent stroke risk and cerebral microbleed burden in ischemic stroke and TIA: a meta-analysis. Neurology (2016) 87(14):1501–10. 10.1212/WNL.0000000000003183 27590288 PMC5075978

[8] Shih AY Blinder P Tsai PS Friedman B Stanley G Lyden PD The smallest stroke: occlusion of one penetrating vessel leads to infarction and a cognitive deficit. Nat Neurosci (2013) 16:55–63. 10.1038/nn.3278 23242312 PMC3952571

[9] Hinman JD Lee MD Tung S Vinters HV Carmichael ST. Molecular disorganization of axons adjacent to human lacunar infarcts. Brain (2015) 138:736–45. 10.1093/brain/awu398 25614025 PMC4339777

[10] Kuba H Adachi R Ohmori H. Activity-dependent and activity-independent development of the axon initial segment. J Neurosci (2014) 34:3443–53. 10.1523/JNEUROSCI.4357-13.2014 24573300 PMC6795309

[11] Gulledge AT Bravo JJ. Neuron morphology influences axon initial segment plasticity. eNeuro (2016) 3(1):85–115. 10.1523/ENEURO.0085-15.2016 27022619 PMC4756267

[12] Rosenbluth J. Abnormal axoglial junctions in the myelin-deficient rat mutant. J Neurocytol (1987) 16:497–509. 10.1007/BF01668504 3681351

[13] Suzuki A Hoshi T Ishibashi T Hayashi A Yamaguchi Y Baba H Paranodal axoglial junction is required for the maintenance of the Nav1.6-type sodium channel in the node of Ranvier in the optic nerves but not in peripheral nerve fibers in the sulfatide-deficient mice. Glia (2004) 46:274–83. 10.1002/glia.20008 15048850

[14] Amor V Feinberg K Eshed-Eisenbach Y Vainshtein A Frechter S Grumet M Long-term maintenance of Na+ channels at nodes of Ranvier depends on glial contact mediated by gliomedin and NrCAM. J Neurosci (2014) 34:5089–98. 10.1523/JNEUROSCI.4752-13.2014 24719088 PMC3983794

[15] Zhou D Lambert S Malen PL Carpenter S Boland LM Bennett V. AnkyrinG is required for clustering of voltage-gated Na channels at axon initial segments and for normal action potential firing. J Cell Biol (1998) 143:1295–304. 10.1083/jcb.143.5.1295 9832557 PMC2133082

[16] Bhat MA Rios JC Lu Y Garcia-Fresco GP Ching W St Martin M Axon-glia interactions and the domain organization of myelinated axons requires neurexin IV/caspr/Paranodin. Neuron (2001) 30:369–83. 10.1016/S0896-6273(01)00294-X 11395000

[17] Sobotzik JM Sie JM Politi C Del Turco D Bennett V Deller T AnkyrinG is required to maintain axo-dendritic polarity in vivo. Proc Natl Acad Sci U S A (2009) 106:17564–9. 10.1073/pnas.0909267106 19805144 PMC2765162

[18] Sun XY Takagishi Y Okabe E Chishima Y Kanou Y Murase S A novel caspr mutation causes the shambling mouse phenotype by disrupting axoglial interactions of myelinated nerves. J Neuropathol Exp Neurol (2009) 68:1207–18. 10.1097/NEN.0b013e3181be2e96 19816196

[19] Amor V Zhang C Vainshtein A Zhang A Zollinger DR Eshed-Eisenbach Y The paranodal cytoskeleton clusters Na+ channels at nodes of Ranvier. Elife (2017) 6:e21392. 10.7554/eLife.21392 28134616 PMC5279941

[20] Okamoto Y Ihara M Fujita Y Ito H Takahashi R Tomimoto H. Cortical microinfarcts in Alzheimer’s disease and subcortical vascular dementia. Neuroreport (2009) 20:990–6. 10.1097/WNR.0b013e32832d2e6a 19483658

[21] Hinman JD. The back and forth of axonal injury and repair after stroke. Curr Opin Neurol (2014) 27:615–23. 10.1097/WCO.0000000000000149 25364952 PMC4459741

[22] Ii Y Maeda M Kida H Matsuo K Shindo A Taniguchi A In vivo detection of cortical microinfarcts on ultrahigh-field MRI. J Neuroimaging (2013) 23:28–32. 10.1111/j.1552-6569.2012.00722.x 22607584

[23] Murphy MP Corriveau RA Wilcock DM Vascular contributions to cognitive impairment and dementia (VCID). Biochim Biophys Acta (2016) 1862:857–9. 10.1016/j.bbadis.2016.02.010 26921818

[24] Li S Carmichael ST. Growth-associated gene and protein expression in the region of axonal sprouting in the aged brain after stroke. Neurobiol Dis (2006) 23:362–73. 10.1016/j.nbd.2006.03.011 16782355

[25] Li S Overman JJ Katsman D Kozlov SV Donnelly CJ Twiss JL An age-related sprouting transcriptome provides molecular control of axonal sprouting after stroke. Nat Neurosci (2010) 13:1496–504. 10.1038/nn.2674 21057507 PMC3059556

[26] Overman JJ Clarkson AN Wanner IB Overman WT Eckstein I Maguire JL A role for ephrin-A5 in axonal sprouting, recovery, and activity-dependent plasticity after stroke. Proc Natl Acad Sci U S A (2012) 109:E2230–9. 10.1073/pnas.1204386109 22837401 PMC3421211

[27] Li S Nie EH Yin Y Benowitz LI Tung S Vinters HV GDF10 is a signal for axonal sprouting and functional recovery after stroke. Nat Neurosci (2015) 18:1737–45. 10.1038/nn.4146 26502261 PMC4790086

[28] Palmer LM Stuart GJ. Site of action potential initiation in layer 5 pyramidal neurons. J Neurosci (2006) 26:1854–63. 10.1523/JNEUROSCI.4812-05.2006 16467534 PMC6793621

[29] Kuba H. Structural tuning and plasticity of the axon initial segment in auditory neurons. J Physiol (2012) 590:5571–9. 10.1113/jphysiol.2012.237305 23027822 PMC3528978

[30] Hedstrom KL Ogawa Y Rasband MN. AnkyrinG is required for maintenance of the axon initial segment and neuronal polarity. J Cell Biol (2008) 183:635–40. 10.1083/jcb.200806112 19001126 PMC2582894

[31] Zhang C Rasband MN. Cytoskeletal control of axon domain assembly and function. Curr Opin Neurobiol (2016) 39:116–21. 10.1016/j.conb.2016.05.001 27203619 PMC4987246

[32] Schafer DP Jha S Liu F Akella T Mccullough LD Rasband MN. Disruption of the axon initial segment cytoskeleton is a new mechanism for neuronal injury. J Neurosci (2009) 29:13242–54. 10.1523/JNEUROSCI.3376-09.2009 19846712 PMC2801423

[33] Stys PK Jiang Q. Calpain-dependent neurofilament breakdown in anoxic and ischemic rat central axons. Neurosci Lett (2002) 328:150–4. 10.1016/S0304-3940(02)00469-X 12133577

[34] Rosenzweig S Carmichael ST. The axon-glia unit in white matter stroke: mechanisms of damage and recovery. Brain Res (2015) 1623:123–34. 10.1016/j.brainres.2015.02.019 25704204 PMC4545468

[35] Sozmen EG Rosenzweig S Llorente IL Ditullio D Machnicki M Vinters HV Nogo receptor blockade overcomes remyelination failure after white matter stroke and stimulates functional recovery in aged mice. Proc Natl Acad Sci U S A (2016) 113(52):E8453–62. 10.1073/pnas.1615322113 27956620 PMC5206535

[36] Hawkes CA Michalski D Anders R Nissel S Grosche J Bechmann I Stroke-induced opposite and age-dependent changes of vessel-associated markers in co-morbid transgenic mice with Alzheimer-like alterations. Exp Neurol (2013) 250:270–81. 10.1016/j.expneurol.2013.09.020 24103194

