# Supplementary figures and images for: Molecular Disorganization of Axons Adjacent to Human Cortical Microinfarcts
Source: Front Neurol. 2017 Aug 16;8:405. doi: 10.3389/fneur.2017.00405 (PMC5561009; doi:10.3389/fneur.2017.00405)

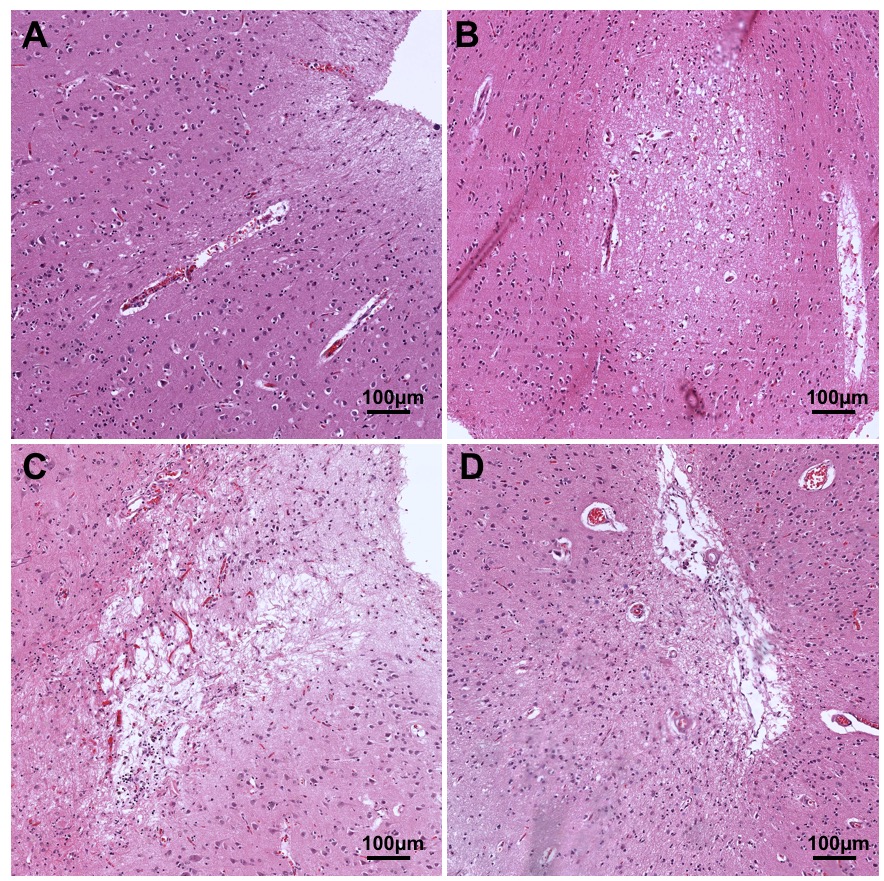

Supplement: Figure S1 — Hematoxylin–eosin stained sections of cortical layers from four patients involved in the study. ×10 magnification. [file image_1.jpeg]

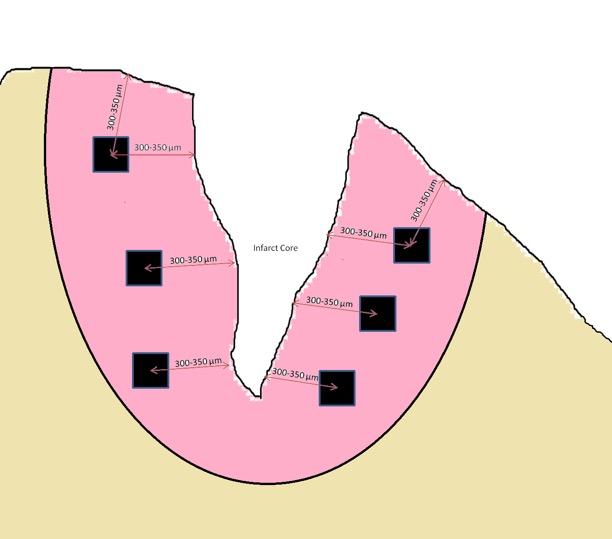

Supplement: Figure S2 — Illustration of a watershed cortical area from which detailed analysis was undertaken. Pink area indicates watershed cortical region with penumbra, and framed areas indicate one-unit area of the sites of the images taken for analysis. Six different areas were examined with equal distance from the infarct core. 1 unit area = 250 µm × 250 µm. [file image_2.jpeg]
